# Quantifying Uncertainty in Pulsed Thermographic Inspection by Analysing the Thermal Diffusivity Measurements of Metals and Composites

**DOI:** 10.3390/s21165480

**Published:** 2021-08-14

**Authors:** Sri Addepalli, Yifan Zhao, John Ahmet Erkoyuncu, Rajkumar Roy

**Affiliations:** 1 School of Aerospace, Transport and Manufacturing, Cranfield University, Cranfield MK43 0AL, UK; yifan.zhao@cranfield.ac.uk (Y.Z.); j.a.erkoyuncu@cranfield.ac.uk (J.A.E.); 2 School of Mathematics, Computer Science and Engineering, University of London, London EC1V 0HB, UK; r.roy@city.ac.uk

**Keywords:** thermal diffusivity, uncertainty quantification, pulsed thermography

## Abstract

Pulsed thermography has been used significantly over the years to detect near and sub-surface damage in both metals and composites. Where most of the research has been in either improving the detectability and/or its applicability to specific parts and scenarios, efforts to analyse and establish the level of uncertainty in the measurements have been very limited. This paper presents the analysis of multiple uncertainties associated with thermographic measurements under multiple scenarios such as the choice of post-processing algorithms; multiple flash power settings; and repeat tests on four materials, i.e., aluminium, steel, carbon-fibre reinforced plastics (CFRP) and glass-fibre reinforced plastics (GFRP). Thermal diffusivity measurement has been used as the parameter to determine the uncertainty associated with all the above categories. The results have been computed and represented in the form of a relative standard deviation (RSD) ratio in all cases, where the RSD is the ratio of standard deviation to the mean. The results clearly indicate that the thermal diffusivity measurements show a large RSD due to the post-processing algorithms in the case of steel and a large variability when it comes to assessing the GFRP laminates.

## 1. Introduction

The last century has seen significant growth in the use of advanced materials including hybrid metallic and composite materials as primary structural components mainly in the aerospace sector. For instance, the need for a low weight, high strength, and damage-resistant material has been the main driver for the adoption of composites into a variety of industrial sectors. These novel and advanced materials offer flexibility and adaptability of the part to set design specifications, which the metallic parts struggle with [1,2]. However, the behaviour and mechanical performance of these materials are not fully understood. Further, the repair of composite parts is a huge challenge when compared with the metallic parts as traditional repair methods may not apply to these, indicating the creation and development of both manufacturing and maintenance systems to support the continuous operability of the system.

The challenge becomes two-fold when it comes to the inspection and evaluation of the structural integrity of the composite parts. Traditional non-destructive testing (NDT) methods such as visual, magnetic particle inspection, and dye penetrant inspection struggle purely due to the heterogeneous, layer-based build nature of the parts. Whilst x-radiography and ultrasonic inspection provide information on the damage, complete characterisation is not available either due to the localised density differences, in which the radiographs add noise and artefacts to, or due to the attenuation of sound when it passes through the material in the case of ultrasound inspection. Though destructive or mechanical testing methods are available, the variability of mechanical material properties such as compressive and tensile strengths, the material hardness, and load bearing capacity, are heavily dependent on the type of material, the fibre geometry (thickness and shape) and weight, the resin matrix, the lay-up/stacking sequence, and the actual design of the final part. All these factors add a huge level of uncertainty in mapping the behaviour of the part, making it a huge challenge for traditional NDT methods to detect damage occurring in these components.

The last decade has seen significant progress in the advancement of pulsed thermography to characterise near and sub-surface damage in composite materials [3,4]. Currently, the process involves an instantaneous optical flash pulse (about 10 milliseconds) that creates a heat transient on a component’s surface and an infrared radiometer captures the surface temperature decay profile over a short period of time, generally over a few seconds [5]. Through image and signal processing algorithms such as the Thermographic Signal Reconstruction (TSR), the decay profile is fitted to deduce a set of time-based reconstructed images that are a true representative of the sub-surface characteristics of the component and are directly dependent on the materials’ thermal diffusivity [3]. With the ability to capture data and represent it with statistical evidence becoming the current trend, the measurement uncertainty still exists especially with the issue translating to not just the data from the radiometer itself but the contributions from the process of inspection, the hardware, or the inspection system together with combinational aspects appearing from data post-processing using a variety of algorithms, each having its own benefit. One of the challenges that has been understood by the scientific community is the inability to map thermal NDT data accurately with a high level of repeatability due to variations in parameters such as the material properties; geometry; and surface finish together with environmental factors such as temperature, humidity, and pressure. All these parameters add to the overall uncertainty in measurement, having an impact on capturing the thermal material property of the composite material.

With concepts such as digital maintenance, design for service, and improvements in computing power, it has become even more important to develop new and powerful tools such as the digital twin that are capable of calculating the remaining useful life based on the current health of the component estimated from the NDT data, together with advanced and predictive data analytics, the sources of which come from the historical and general overall knowledge on the behaviour of the component [6]. Methods such as the principle component analysis, confidence-based data mapping, and the measurement of the actual material property lead to a more accurate level of sentencing the part, ensuring both design conformity and enhancing the reliability of the overall performance of the asset [7,8]. The challenge still remains in providing an understanding of uncertainty that occurs at the process level that makes an impact on the overall measurement itself [9]. Uncertainty is generally referred to as the probability or the statistical distribution of the measured value achieved from the repeatability and reproducibility of the measurement and represented in the form of a standard deviation [10]. The measuring device, in this case a research-grade quantum detector-based radiometer (FLIR SC7600 MB InSb), has the ability to determine the best data-capture parameters together with the best data fitting parameters, and determines the process level combined uncertainty associated with the pulsed thermographic inspection at the system-of-system level. 

This paper primarily uses pulsed thermography technique to determine the thermal diffusivity of both metallic and composite materials and investigates the effect of various parameters that potentially add uncertainty in measurement. For this research, only the following parameter variations have been employed to deduce the process level uncertainty:

- Uncertainty caused by three post-processing algorithms
- Uncertainty caused by flash power
- Uncertainty caused by repeat tests
- Uncertainty caused by multiple samples

## 2. The Fundamentals

In order to calculate and evaluate the values of thermal diffusivity and the relative standard deviation (RSD^*^—the ratio between the standard deviation and the mean of the measured thermal diffusivity), it is necessary to understand the fundamental heat theory together with the governing algorithms. This section provides information on the heat diffusion theory and the post processing algorithms used to deduce the values. In this paper, the RSD is given by

(1) $$RSD^{*}=\frac{Standard\;Deviation}{Mean\;Diffusivity}$$

### 2.1. Heat Diffusion Theory

**Figure 1:**
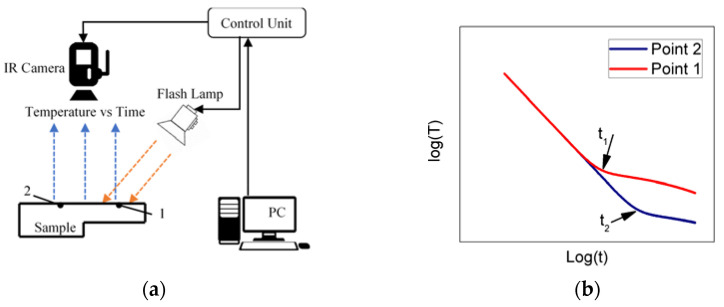
(**a**) Pulsed thermography experimental setup; 1 denotes a position on the sample surface with a reduced thickness and 2 denotes the through-thickness of the sample. (**b**) Typical observed time-temperature decay curves in the logarithmic domain for points 1 and 2, respectively, where the time of heat saturation $t_{1}$ and $t_{2}$ are the keys to measure the thickness of the material.

In pulsed thermographic inspection, a short and high energy light pulse is projected onto the sample surface through flash lamps (Figure 1a). Heat conduction then takes place from the heated surface to the interior of the sample, leading to a continuous decrease in the surface temperature of the sample, which is captured using a PC-controlled infrared camera [11] (see Figure 1b). When this time-dependent decay profile data is plotted for areas of different thicknesses, heat saturation represented by change in slope occurs when the heat wave reaches the backwall of the sample. The thermal diffusivity can then be calculated based on the time when the temperature deviation occurs and the thickness of the material itself.

As shown in Figure 1b, if thickness is known, the thermal diffusivity for point 1 and 2 on the surface can be estimated based on the time of temperature deviation, $t_{1}$ and $t_{2}$, respectively. The surface temperature due to the back-wall at depth L for a homogeneous plate is given by [12]

(2) $$T\left(0,\;t\right)=\frac{Q}{\sqrt{\pi \rho ckt}}\left[1+2\overset{\infty }{\underset{n=1}{\sum }}R^{n}\exp\left(-\frac{n^{2}L^{2}}{\alpha t}\right)\right]$$

where T(0, t) is the temperature variation of the surface at time t, Q is the pulse energy, ρ is the material density, c is the heat capacity, k is the thermal conductivity of the material, R is the thermal reflection coefficient of the air gap interface, and α is the thermal diffusivity.

### 2.2. Data Post-Processing Algorithms

To illustrate the working behaviour and the characteristic changes in the measurement due to the selection of an appropriate post-processing algorithm, three methods were chosen: log second derivative (LSD) method, absolute peak slope time (APST) method, and new least-squares fitting (NLSF) method. The following sub-sections provide the fundamental equations for the three methods.

#### 2.2.1. Log Second Derivative (LSD) Method

A linear relation in the logarithmic domain with slope −0.5 as Equation (3) exists for time and temperature if both sides of Equation (2) are applied by the logarithmic operation [13]

(3) $$\ln\left[T\left(t\right)\right]=\ln\left[\frac{Q}{\sqrt{\pi \rho ckt}}\right]-0.5\ln\left(t\right)$$

The temperature response for any change in the thermal material property from the structure, damage, or defect will deviate from the linear response. Shepard [14] proposed the Thermal Signal Reconstruction (TSR) technique to reduce temporal noise using a high order polynomial model to fit the temperature cooling curve. The model can be written as

(4) $$\ln\left[T\left(t\right)\right]=\overset{N}{\underset{i=0}{\sum }}a_{i}{\left[\ln\left(t\right)\right]}^{i}$$

where T(t) is the surface temperature at time t, N is the model order, and $a_{i}$ are the coefficients to be estimated. Once the unknown coefficients $a_{i}$ are estimated by the least square method, the temperature behaviour can be reconstructed to replace the raw data. The first and second derivatives of ln[T(t)] with respect to ln(t) can be calculated from the estimated coefficients directly, and are expressed as

(5) $$\frac{d\ln\left[T\left(t\right)\right]}{d\ln\left(t\right)}=\overset{N}{\underset{i=1}{\sum }}a_{i}\cdot i\cdot {\left[\ln\left(t\right)\right]}^{i-1}$$

(6) $$\frac{d^{2}\ln\left[T\left(t\right)\right]}{d\ln^{2}\left(t\right)}=\overset{N}{\underset{i=2}{\sum }}a_{i}\cdot i\cdot \left(i-1\right)\cdot {\left[\ln\left(t\right)\right]}^{i-2}$$

The advantage of using such a parametric method is that the noise can be significantly reduced when calculating the first or second derivatives through looking at model coefficients rather than the raw data itself. 

Shepard [14] proposed a Log Second Derivative (LSD) method to estimate the depth of a defect or the thickness of a sample by

(7) $$L=\sqrt{t_{LSD}\cdot \pi \cdot \alpha }$$

where $t_{LSD}$ is the peak time of the second derivative, and α is the calculated thermal diffusivity.

#### 2.2.2. Absolute Peak Slope Time (APST) Method

Zeng et al. [15,16], proposed to first multiply both sides of Equation (2) with $\sqrt{t}$, and define a new time-dependent function f(t) as

(8) $$f\left(t\right)=T\left(t\right)\cdot \sqrt{t}=\frac{Q}{e\sqrt{\pi }}\left[1+2\overset{\infty }{\underset{n=1}{\sum }}\exp\left(-\frac{n^{2}L^{2}}{\alpha t}\right)\right]$$

where $e=\sqrt{\rho ck}$ is the thermal effusivity, and $R^{n}$ is neglected by assuming *R* = 1. The first derivative of f(t) is then expressed as

(9) $$f^{\prime }\left(t\right)=\frac{2Q}{e\sqrt{\pi }}\left[\overset{\infty }{\underset{n=1}{\sum }}\exp\left(-\frac{n^{2}L^{2}}{\alpha t}\right)\cdot \frac{n^{2}L^{2}}{\alpha t^{2}}\right]$$

The peak time of $f^{\prime }\left(t\right)$, $t_{APST}$, is the corresponding time that the second derivative of f(t) equals to zero, expressed as

(10) $$f^{\prime \prime }\left(t_{APST}\right)=\frac{2Q}{e\sqrt{\pi }}\left[\overset{\infty }{\underset{n=1}{\sum }}\exp\left(-\frac{n^{2}L^{2}}{\alpha t_{APST}}\right)\cdot \frac{n^{2}L^{2}}{\alpha t_{APST}{}^{3}}\cdot \left(\frac{n^{2}L^{2}}{\alpha t_{APST}}-2\right)\right]=0$$

and the solution can be written as

(11) $$t_{APST}=\frac{L^{2}}{2\alpha }$$

When multiple reflections are considered, the equation can also be expressed as [16]

(12) $$t_{APST}=\frac{L^{2}}{1.93\alpha }$$

to provide a more accurate estimation. Knowing the value of L and $t_{APST}$ determines α.

#### 2.2.3. New Least-Squares Fitting (NLSF) Method

Finding a characteristic time to correlate with defect depth or thickness provides the basis for both methods expressed above. As both the LSD and the APST methods are based on fitting polynomial models without considering the physical principle of heat diffusion, the processes are vulnerable to signal corruption due to a high-level noise being introduced into the data. Conversely, methods using curve fitting based on the heat-diffusion model have been used in laser-flash diffusivity methods [17] and are typically not susceptible to data noise.

To address the above limitations, Zhao et al. [8] introduced an analytical model aiming to not only estimate the depth more accurately but also measure the thermal wave reflection coefficient. The proposed analytical model is written as

(13) $$\tilde{T}\left(t,B,\;W,R,t_{s},s\right)=\frac{B}{\sqrt{t+t_{s}}}\left[1+2\overset{M}{\underset{n=1}{\sum }}R^{n}\exp\left(-\frac{n^{2}W}{t+t_{s}}\right)\right]-s\left(t+t_{s}\right)$$

where $B=\frac{Q}{\sqrt{\pi \rho ck}}$, $W=\frac{L^{2}}{\alpha }$, *t_s_* is the starting time of sampling, and M is a large iteration number. The introduction of $t_{s}$ is unique as it allows the proposed method to be applicable to any segment of the collected data. There are five parameters to be estimated in this case, and include $t_{s}$, R, W, B, and s. The nonlinear least-squares solver in Matlab (lsqnonlin) is employed to solve these five-parameters’ optimisation problem. Through initially setting the lower and upper bounds for each parameter, this method estimates the optimal parameters that has

(14) $$\underset{B,WR,t_{s},s\;}{\min}\left\|\tilde{T}\left(t\right)-T\left(t\right)\right\|$$

The initial value of the parameter $t_{s}$ is selected as zero and the lower and upper bounds are selected as -1 and 1, respectively, because it is usually very small. The initial value of R is selected as 1 and the lower and upper bounds are selected as 0 and 1. The selection of B depends on the energy applied on the inspection surface, and the selection of W depends on the material and the thickness of the samples (estimated by $W=\frac{L^{2}}{\alpha }$). The lower and upper bounds of W and A are usually selected as five times lower and five times higher than the initial values. The lower and upper bounds of s are selected as −50 and 50, and the initial value is chosen as 0. It should be noted that the computational time for this method depends on the selection of the initial value together with the lower and upper bounds.

Once the optimal parameters are estimated, for a known α, the thickness can be estimated by

(15) $$L=\sqrt{W\cdot \alpha }$$

Alternatively, if L is known, the thermal diffusivity can be estimated by

(16) $$\alpha =\frac{L^{2}}{W}$$

## 3. Methods & Materials

This section mainly describes the experimental setup, the target materials, and the inspection scenarios to evaluate RSD as a value of uncertainty.

### 3.1. Pulsed Thermography

The pulsed thermography system used for this research is a commercial system that goes by the trade name Thermoscope II^®^ supplied by Thermal Wave Imaging Inc., Michigan, USA. The system consists of

- a FLIR 7600 MB radiometer, which is a 640 × 512 pixels, cooled, indium antimonide (InSb)-based quantum detector;
- a capacitor bank-powered, twin xenon flash lamp that has a nominal overlap power at source of 25 KJ (or 2 KJ (nominal) for a sample surface area of 250 × 200 mm) enclosed in a highly reflective box hood;
- a computer control unit that controls the flash unit and captures data from the radiometer using the Mosaiq^®^, which is supplied with Thermoscope II^®^

The working principle is as described in Section 2.1.

### 3.2. Materials

For this paper, four publicly available materials with known thermal diffusivities (provided by material suppliers) were selected;

- aluminium (Al98, diffusivity—79.4 mm^2^/s) and mild steel (19.7 mm^2^/s) (to represent metals)
- carbon fibre-reinforced polymer (CFRP) (0.48 mm^2^/s) and glass fibre-reinforced polymer (GFRP) (0.58 mm^2^/s) (to represent composite materials)

Whilst the metal samples were of the dimension 150 × 150 × 8 mm, the composites were cut to the dimension of 150 × 100 × 4 mm. For the composite materials, uni-directional fibres in an epoxy prepreg were laid up in a quasi-isotropic stacking sequence. Also, for each sample type, a set of three repeat samples were used for this work to achieve values of statistical significance. Whilst the metallic samples were coated with Graphit33^®^ soot spray to improve the emissivity, the composite materials were inspected in the ‘as-manufactured’ state.

### 3.3. Inspection Scenarios

In order to demonstrate the uncertainties arising from a variety of parameters, the following four scenarios were used for the measurement of the materials’ thermal diffusivity:

- Multiple algorithms—three post processing algorithms: LSD, APST, and NLSF were used to calculate the thermal diffusivity (Section 2.2)
- Flash power—for the pulsed thermography inspection, four preset flash powers of 25%, 50%, 75%, and 100% (maximum equivalent to 25 KJ for 100% setting) were used, these power settings are controlled by the Mosaiq^®^ software
- Repeat tests—a set of three repeat tests for the same sample for each of the four materials were performed to capture differences due to the actual inspection itself,
- Multiple samples—the same experimental capture parameters were used to capture data from three independent samples from the four material types: aluminium, mild steel, CFRP, and GFRP.

Two different data capture settings were used to match the thermal diffusivities of the target samples. In order to capture the diffusivity for metals, them having a higher rate of heat diffusion, the camera acquisition frame rate was set to 50Hz with a 5 s data capture length, which was evaluated to be the time required for the thermal wave to reach the backwall of the 8-mm samples (model computed using Equations (3) and (4)) [3]. The data acquisition frame rate was set to 25 Hz with a 12 s data capture length to match the slower diffusivity values of the composite materials. In both cases, the flash pulse length was set at 10 milliseconds.

## 4. The Findings

As presented in the previous sections of this paper, the results obtained under the four scenarios are presented in this section. An overall discussion of the results is provided in the next section to tie up all the findings from this research.

### 4.1. Uncertainty from Three Post-Processing Algorithms

Acquiring the data in a universal RAW format may be the easiest of processes but reconstructing the data to extract meaningful information is complicated. Each of the algorithms, LSD, APST, and NLSF come with their own characteristics, which leaves the end user to make a selection appropriate to his requirement. It must be noted that the aim of this paper is not to compare the different algorithms but more so to present the measurement uncertainties that arise due to their applicability, especially when the dataset is the same in all the cases presented below.

**Figure 2:**
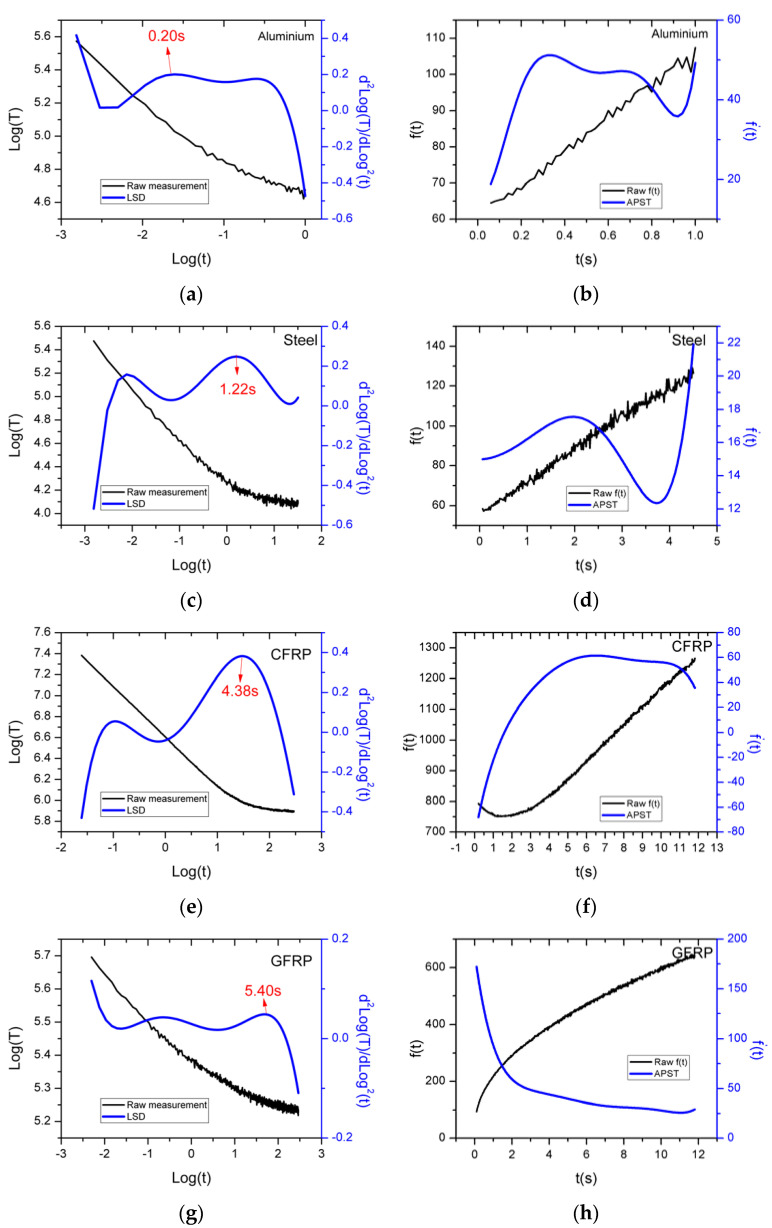
Left: (**a**,**c**,**e**,**g**) RAW thermal plots in the log domain (black curves) and the second derivative plots for aluminium, steel, CFRP, and GFRP plotted using LSD method (blue curves). Right: (**b**,**d**,**f**,**h**) The black curves indicate the raw f(t) in Equation (8) in the time domain, and the blue curves indicate the first derivative of f(t) plotted using the APST method for aluminium, steel, CFRP, and GFRP.

To demonstrate the representation of the thermal decay profile of the material extracted from the RAW data by the post-processing algorithms, the LSD and APST methods were chosen to show the difference in how they plot the data. The set of plots on the left side in Figure 2 below show the thermographic signal reconstruction (TSR) data plot, showing the RAW profile (represented by the black curve) and the second derivative peak (blue curve) plotted using the LSD method for the four materials. Similarly, the right-hand side plots seen in Figure 2 represent the plot for the function f(t) (Equation (8)) (black curve) against the first derivative of f(t) (blue curve) plotted using the APST method. As seen from Figure 2, it can be noticed that, due to the plotting nature of the algorithms, the RAW profiles are completely different for the ‘as-is’ and f(t) data for all the materials, still representing the same data. To fully understand the capability of the algorithms, the second derivative plot using LSD and the first derivative plot for the APST were selected.

As expected, the second derivative peak is picked up by the LSD for all the materials with the profile and thermal intensity being directly proportional to the material property itself. However, when a similar reconstruction for a first derivative plot is deployed using the APST method, the algorithm struggles to produce a strong pattern mainly for the GFRP sample, which is represented in Figure 2 as a decreasing APST curve failing to deduce the diffusivity of that particular laminate. This was found to be the case for multiple repeats, indicating the varied nature of the GFRP material itself. When compared with the second derivative peak, GFRP still struggled to produce a strong result, confirming the thermal attenuation of the material.

In order to understand the differences in the calculated thermal diffusivity values, the raw data acquired from each of the materials was reconstructed based on the equations mentioned in Section 2.2 and the results are tabulated in Table 1. Whilst GFRP and Steel show maximum RSD of 20.2% and 19%, respectively, they are in line with the thermal behaviour of the material itself. The CFRP shows the lowest difference from the algorithms’ performance, with GFRP not producing any diffusivity value from the APST method due to its negative decay profile.

**Figure 3:**
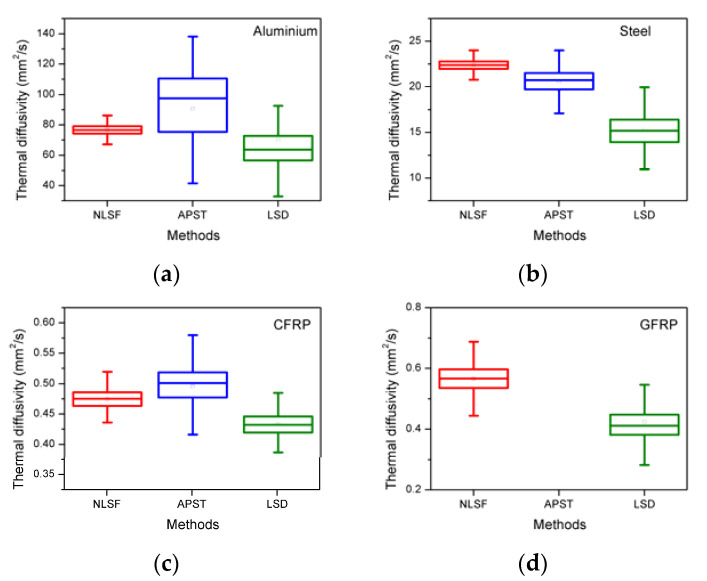
Box charts of the estimated thermal diffusivity (y-axis) for four materials (**a**) Aluminium, (**b**) Steel, (**c**) CFRP, and (**d**) GFRP using the LSD, APST, and NLSF methods (x-axis).

Figure 3 shows the box charts of the estimated thermal diffusivity measurements for all the materials under study reproduced by applying all three: LSD, APST, and NLSF post-processing algorithms.

In general, the NLSF algorithm seems to have produced a more representative estimated diffusivity measurement range in comparison with the LSD and APST methods. Further, the APST method produced a noticeable variability in the diffusivity measurement for three of the four materials with failure to produce the measurement for the GFRP material. This is due to the thermal material property of GFRP and not just the uncertainty associated with the post-processing algorithm used. Due to the lowest uncertainty range and its suitability to process all samples, the NLSF algorithm is used for calculating the thermal diffusivity for the remainder of this paper [18].

### 4.2. Uncertainty from Variable Flash Power

**Figure 4:**
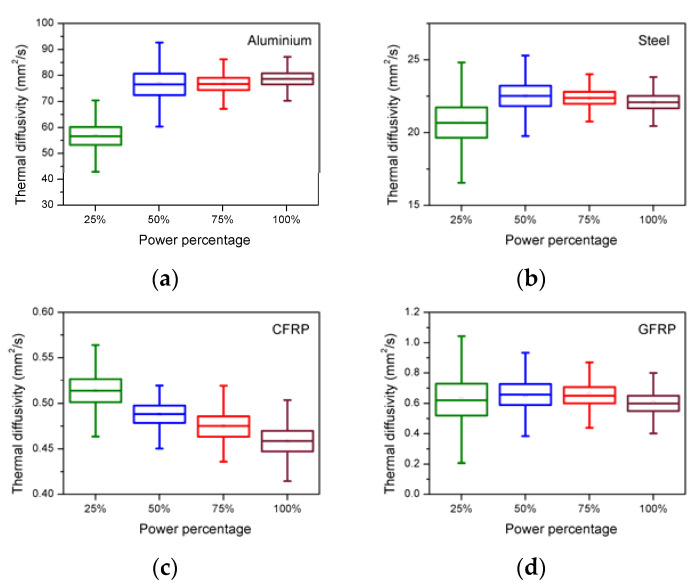
Box charts of the estimated thermal diffusivity (y-axis) with different power percentages (x-axis) for four materials (**a**) Aluminium, (**b**) Steel, (**c**) CFRP, and (**d**) GFRP using the NLSF method.

As mentioned in Section 3.3, four preset flash powers at 25%, 50%, 75%, and 100% were used to perform the pulsed thermography inspection on all four materials, and the thermal diffusivities of each of the materials at each identified flash power setting were calculated using the NLSF method. The box charts (Figure 4) and the tabulated (Table 2) measurements of the diffusivities of the four materials were determined in the same manner as shown in Section 4.1.

It is clear from the Table 2 that, for the variation of the power settings, the RSD for Steel, CFRP, and GFRP is at 3.7%, 4.7%, and 3.9%, all calculated using the NLSF method. This indicates that the variation due to flash power has the least impact for the three materials with aluminium becoming the exception, showing a large variation represented with an RSD of 14.4%. This is probably due to the materials’ ability to absorb the instantaneous heat and the sensor’s inability to capture the response at that rate, which mainly contributes to the uncertainty in this case. A closer observation indicates that the composites perform well even under lower energy exposure due to their lower thermal conductivity, which has already been established by multiple researches [19,20]. As a rule of thumb, it is better to set the inspection flash energy exposure to at least 1.5 KJ (nominal), which is equivalent to 75% preset on the flash system and above for composite materials and a full 2 KJ (nominal)/100% for metals purely to achieve the depth of penetration that is in line with the literature [3,18,21].

### 4.3. Uncertainty due to Repeated Tests

As indicated in the inspection scenario in Section 3.3, to identify the uncertainty caused due to multiple tests, three individual inspections for repeatability were performed on the same (single) sample for each of the four materials, and the thermal diffusivity measurements are computed and presented in Table 3. Table 3 illustrates that the variability between tests performed on the same sample belonging to each of the four material types seems be minimal in comparison with that caused due to the application of the algorithm and the flash power, indicating that the uncertainty for repeat tests is minimal. 

Additionally, as indicated previously, GFRP shows the largest variation with a measured RSD of 3.4%, followed by aluminium at 1.8%, indicating the impact of the material property of those materials. 

### 4.4. Uncertainty Caused by the Sample

The last of the inspection scenarios, a set of three samples from the same material type, in this case, samples cut from the large plate, was subjected to the pulsed thermographic inspection. The raw data from the inspection was then post-processed using the NLSF algorithm to deduce the thermal diffusivity of each of the samples and is tabulated below (Table 4).

The trend in this case differs, showing higher variability for both CFRP and GFRP when compared with that of the metal samples. Whilst the largest variability is caused by GFRP (RSD—8.3%), the lowest comes from steel (RSD—1.8%). This is primarily due to the fact that metals in general show a larger homogeneity with both CFRP and GFRP, representing the heterogeneous nature of the laminates that contributed due to the multiple layers of the material. Further, it must be acknowledged that the composite materials in this case were hand laid using a prepreg material, adding manufacturing variability, causing the difference in the localised fibre volume ratio.

## 5. Discussion

**Figure 5:**
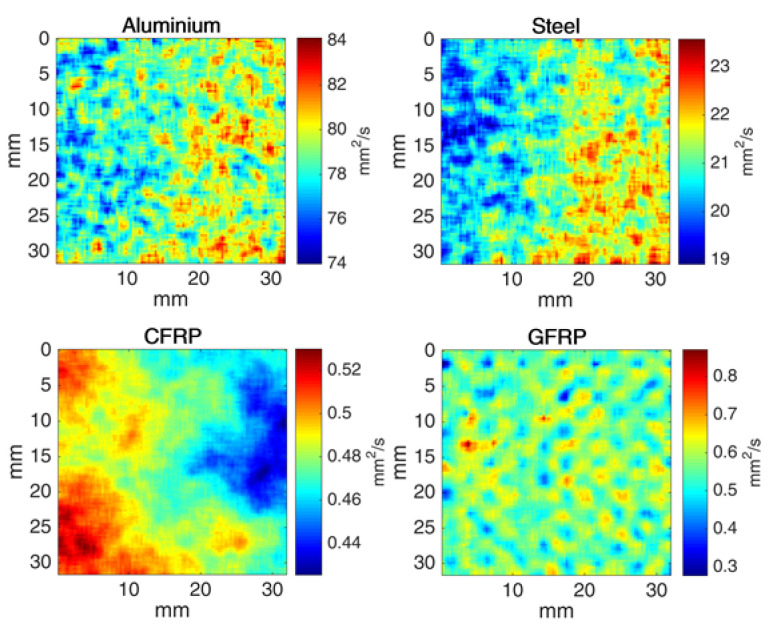
Thermal diffusivity distribution map representing one selected sample from all four materials using the NLSF method.

The results section presented the direct measurement of thermal diffusivity and introduced the common metric of relative standard deviation or RSD as a measure for uncertainty caused due to the four scenarios: post-processing algorithms, flash power, repeat tests, and multiple samples. As part of this research and based on the initial variability due to multiple algorithms, the NLSF method was selected for the rest of the analysis presented in this paper. Whilst actual measurements were presented as values and plots, the data was also reconstructed as a diffusion map as illustrated in Figure 5 below, to be in line with the previous research [22].

The thermal diffusivity map, illustrated by Figure 5, shows that the metallic samples have larger diffusion values when compared with that of the composite materials, confirming the differences in their thermal conductivity. The patterns are representative of the actual measurements calculated using the NLSF method.

**Figure 6:**
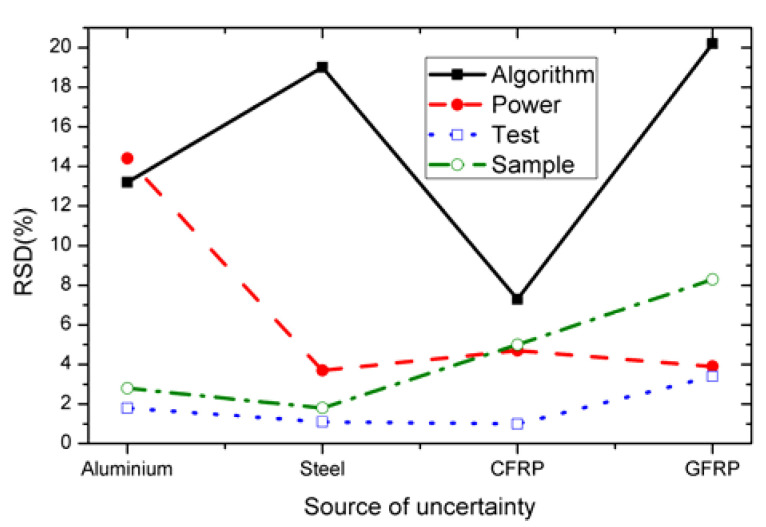
Uncertainty comparison caused by various sources for the considered four materials.

The results section clearly presented that the largest variability based on the RSD values came directly from the deployment of the post-processing algorithms itself. To make the results more understandable, the RSD values acquired from the use of multiple algorithms for all the inspection scenarios were plotted (Figure 6). It can be inferred from the comparison plot (Figure 6) that the largest uncertainty comes from the choice of the algorithm itself followed by the flash power, material, and the repeat tests. 

**Figure 7:**
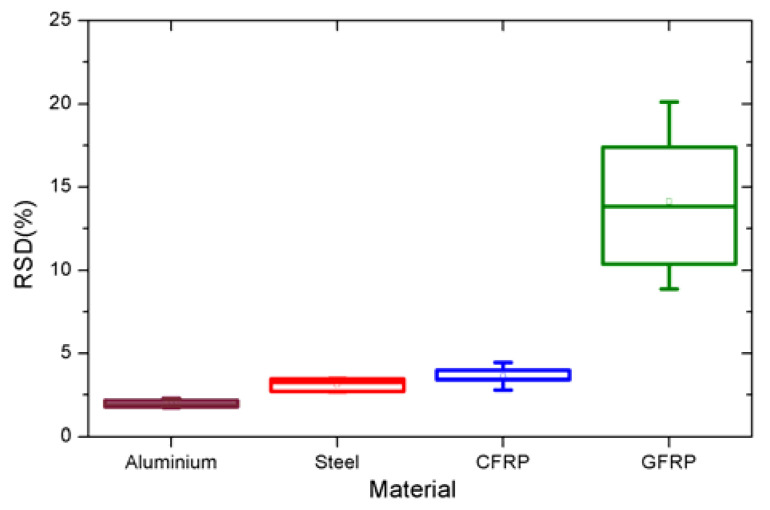
The variation of the estimated thermal diffusivity of a sample based on five tests of each material (three tests for the same sample and two tests for two other different samples).

Based on the insights from Figure 6, a secondary statistical analysis was performed and the RSD value deduced from multiple materials was replotted (Figure 7), this parameter of material type contributing to the second highest uncertainty.

The secondary statistical analysis combined the inspection data for all the materials at 100% flash and included the three repeat datasets together with the two additional datasets obtained from each individual sample belonging to the same type of material. The new insights revealed that the largest variability came from the type of material being inspected. In this case, GFRP was found to have the maximum variability through this alternate analysis, indicating the issues associated with this particular type of material. This is largely due to the non-conductivity and the reflectivity of the glass fibre material itself with only the resin matrix delivering the absolute value. This also now explains the inability of the APST being unable to produce a positive thermal diffusivity value, which is evidenced by the results presented above. It must be understood that both the choice of algorithms and the type of material being inspected contribute to the overall uncertainty.

## 6. General Guidelines for Pulsed Thermography Inspection

A large part of the academic research in the area of pulsed thermography is focused on a variety of aspects such as detectability and applicability of the inspection technique to a variety of materials, geometries, damage features, data fitting, and even to the extent of measuring material properties. The industry on the other hand requires a confidence-based inspection system that produces results with high fidelity, repeatability, reliability, and accuracy. This research has clearly shown that the uncertainty associated with the inspection techniques is primarily dependent on two attributes, the post-processing algorithm deployed and the fundamental material property itself. Based on the above results, the authors propose the following guidelines:

- Step 1: Evaluate the material being subjected to inspection. This evaluation should include not just the material type but also the geometry and surface finish of the part that is being inspected.
- Step 2: Perform a preliminary analysis on the material, setting the capture parameters in terms of flash energy and data length for a target component.
- Step 3: Select a suitable post-processing algorithm to post-process the inspection data. Based on the current research, NLSF seems to be an all-rounder, showing both reliability and accuracy of the final result produced.
- Step 4: Establish a baseline uncertainty test that can then be added to the actual result.

It is envisaged that these guidelines will not just improve the deployability of the inspection technique but the overall reliability of the inspection data where the actual strength of the thermal non-destructive evaluation lies. It must be understood that, for the purpose of this research, not all factors contributing to uncertainty were considered and must be evaluated on a case-by-case basis.

## 7. Conclusions

This paper presents a novel method of deducing uncertainty of the pulsed thermography inspection process in terms of standard deviation, established through this paper as the relative standard deviation or RSD. As part of this research, thermal diffusivity values were used as a measure to identify major sources of uncertainty arising from the use of multiple algorithms, the impact of flash power, and the effect of multiple materials and repeat tests. The results show that the repeat tests show the lowest uncertainty with the major sources coming from the actual post-processing algorithm deployed and the material property itself with little or no impact from the experimental parameters, assuming that these were established in line with the theory represented by Equations (3) and (4). The statistical analysis independent of the type of material shows that the selection of the post-processing algorithm will contribute to a larger level of measurement uncertainty. The results also show that the NLSF algorithm shows a better performance in comparison with the LSD and APST. A further analysis also revealed that the GFRP has the largest uncertainty, which is directly due to its intrinsic material property. It is envisaged that using the pulsed thermography inspection with the recommended guidelines will help improve the accuracy and reliability of the inspection.

## Figures and Tables

**Table 1:** Measured thermal diffusivity (mm^2^/s) values with RSD* using the three methods.

Method	Aluminium	Steel	CFRP	GFRP
NLSF	76.74 ± 3.63	22.40 ± 0.61	0.47 ± 0.02	0.57 ± 0.06
APST	90.82 ± 23.33	20.60 ± 1.53	0.50 ± 0.03	
LSD	70.41 ± 35.29	15.29 ± 1.82	0.43 ± 0.02	0.42 ± 0.07
Overall	79.33 ± 10.45	19.43 ± 3.70	0.42 ± 0.03	0.49 ± 0.10
RSD*	13.2%	19.0%	7.3%	20.2%

**Table 2:** Measured thermal diffusivity (mm^2^/s) values with RSD for different power percentages using the NLSF method.

Power	Aluminium	Steel	CFRP	GFRP
25%	56.80 ± 5.19	20.74 ± 1.56	0.51 ± 0.02	0.63 ± 0.18
50%	76.81 ± 5.96	22.52 ± 1.04	0.49 ± 0.01	0.65 ± 0.13
75%	76.74 ± 3.63	22.40 ± 0.61	0.47 ± 0.02	0.66 ± 0.11
100%	79.01 ± 6.13	22.12 ± 0.59	0.46 ± 0.02	0.60 ± 0.11
Overall	72.34 ± 10.41	21.95 ± 0.82	0.49 ± 0.02	0.64 ± 0.03
RSD	14.4%	3.7%	4.7%	3.9%

**Table 3:** Measured thermal diffusivity (mm^2^/s) values with RSD for multiple tests of the same sample using the NLSF method.

Test	Aluminium	Steel	CFRP	GFRP
Test 1	78.91 ± 1.35	21.07 ± 0.74	0.48 ± 0.02	0.60 ± 0.12
Test 2	76.08 ± 1.75	21.50 ± 0.74	0.47 ± 0.02	0.56 ± 0.05
Test 3	77.68 ± 1.68	21.53 ± 0.58	0.47 ± 0.01	0.59 ± 0.06
Overall	77.56 ± 1.42	21.37 ± 0.25	0.47 ± 0.01	0.59 ± 0.02
RSD	1.8%	1.1%	1.0%	3.4%

**Table 4:** Measured thermal diffusivity (mm^2^/s) values with RSD for multiple samples using the NLSF method.

Samples	Aluminium	Steel	CFRP	GFRP
Sample 1	78.91 ± 1.35	21.07 ± 0.74	0.48 ± 0.02	0.60 ± 0.11
Sample 2	76.10 ± 1.36	21.73 ± 0.58	0.44 ± 0.02	0.61 ± 0.08
Sample 3	74.58 ± 1.41	21.82 ± 0.71	0.44 ± 0.02	0.63 ± 0.13
Overall	76.53 ± 2.20	21.54 ± 0.41	0.45 ± 0.02	0.61 ± 0.05
RSD	2.8%	1.8%	5%	8.3%

## Data Availability

For access to the data underlying this paper, please see the Cranfield University repository, CORD, at DOI: 10.17862/cranfield.rd.11919987.
